# Supplementary material for: Structure-guided engineering of α-ketoisocaproate dioxygenase increases isobutene production in Synechocystis sp. PCC 6803
Source: Microb Cell Fact. 2025 Apr 23;24:93. doi: 10.1186/s12934-025-02708-x (PMC12020224; doi:10.1186/s12934-025-02708-x)
Supplement: Supplementary file 1 — Additional file 1. [file 12934_2025_2708_MOESM1_ESM.docx]

**Supplementary information for Structure-guided engineering of α-ketoisocaproate dioxygenase increases isobutene production in *Synechocystis* sp. PCC 6803**

Conrad Schumann†, Amit Kugler†, Bhavik Ashwin Shah, Gustav Berggren, Henrik Land, Cecilia Blikstad, Karin Stensjö*

Department of Chemistry-Ångström Laboratory, Uppsala University, SE-751 20, Uppsala, Sweden

*Corresponding author:

† These authors contributed equally.

**Supplementary Figure 1**


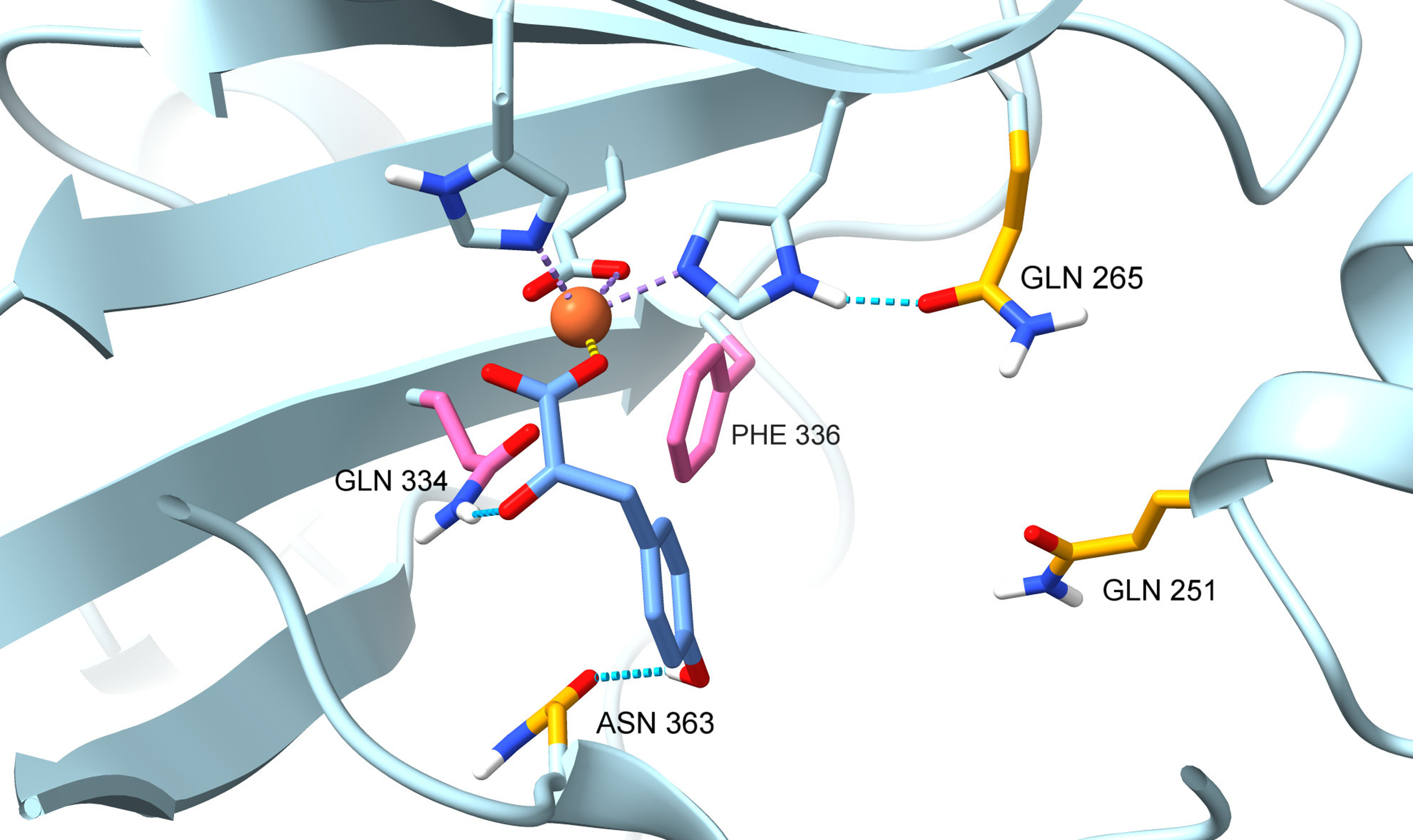


**Supplementary Figure 1** Structural view of the *Rn*KICD homology model active site in open conformation with HPP as substrate. Rational design targets are highlighted in yellow, while site-saturation targets are marked in pink. The structure of HPP is visualized in blue.

**
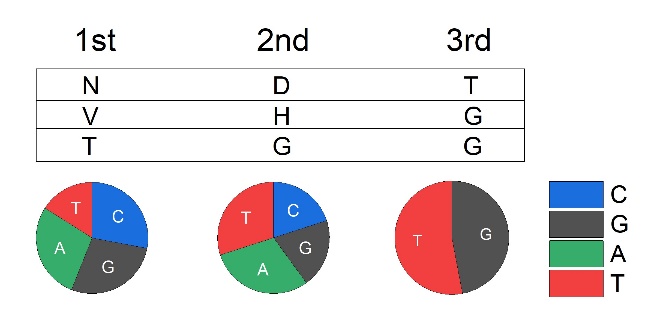

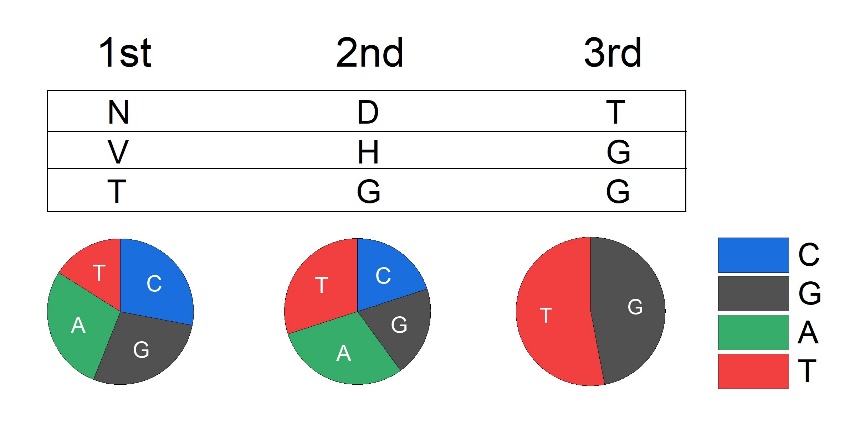
Supplementary Figure 2**

**A**


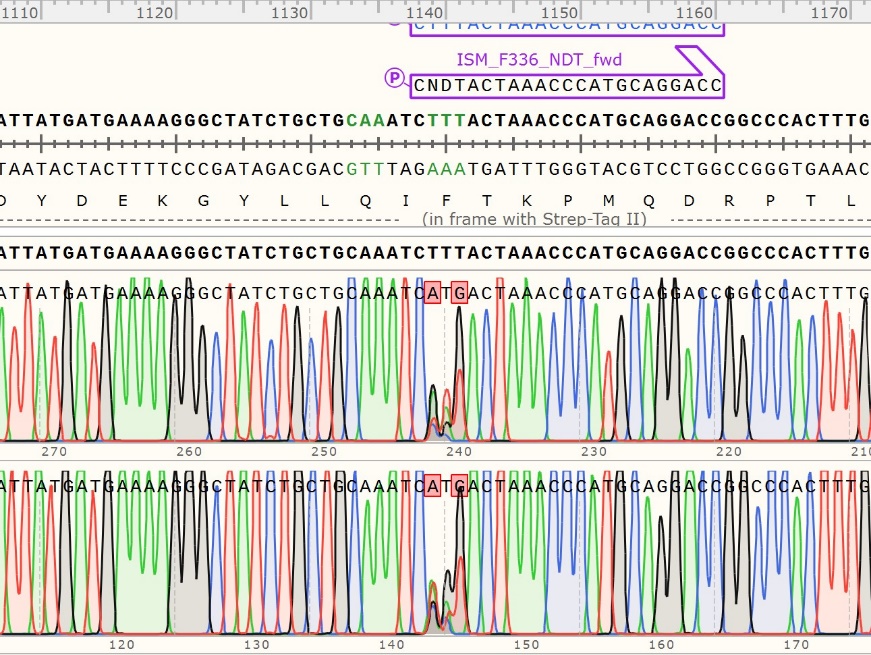

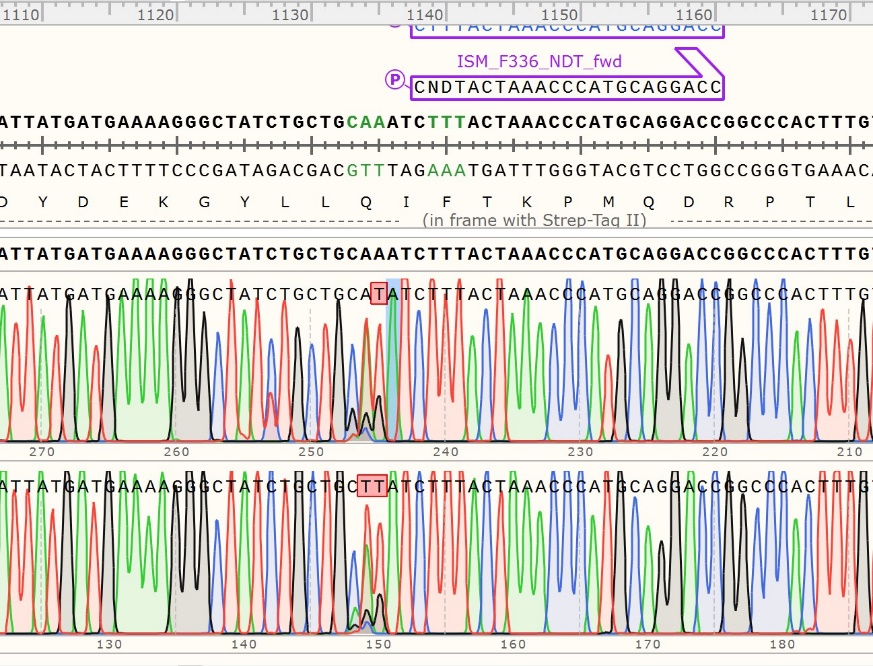

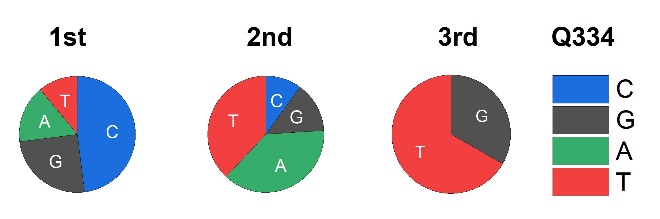

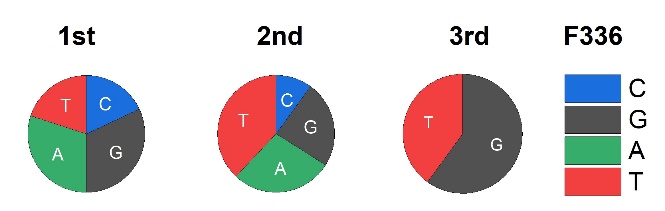


**C**

**B**

**Supplementary Figure 2** Quick quality control (QQC) of libraries generated by site-saturation mutagenesis. A. Degenerate codons and the expected codon distribution according to Kille et al., 2013. B. QQC of site-saturation mutant library on Q334 codon with Sanger sequencing result above and nucleobase distribution below. B. QQC of site-saturation mutant library on F336 codon with Sanger sequencing result above and nucleobase distribution below. The pie charts show the nucleobase distribution in all three codon positions based on estimations from the sequencing results (peak height in the three codon positions). Blue, cytosine/C; black, guanine/G; green, adenine/A; red = thymine/T. The overlapping sequencing result in B (position 253) is most likely an error or artifact in the sequencing of the pooled library plasmid. Later sequencing results of the identified hits did not show a deviation from the WT sequence in position 253 encoding for L332. CTG and TTG would also both encode for leucin making the potentially observed mutation silent.

**Supplementary Figure 3**


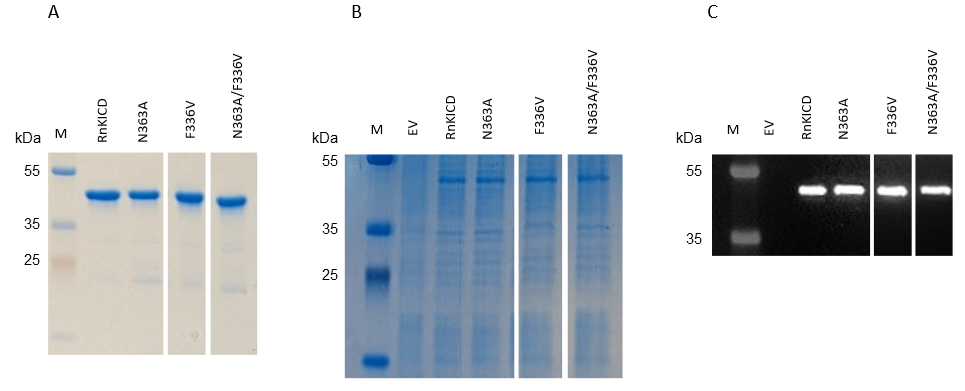


**Supplementary Figure 3** A. SDS-PAGE of purified *Rn*KICD variants. B. SDS-PAGE analysis of recombinant RnKICD variants extracted from *Synechocystis*. C. Western blot analysis of recombinant RnKICD variants extracted from *Synechocystis*. EV is referred to as a *Synechocystis* strain expressing pEEK2 empty vector. M, protein marker.

**Supplementary Figure 4**

**Supplementary Figure 4**. *In vitro* isobutene production in presence of competing substrate HPP and comparison of concentration effects quantified via GC-MS. Reaction mixture was supplemented with 3mM KIC and 10 or 1000µM of HPP and the reaction was initiated by addition of the respective purified enzyme. Isobutene production without HPP was considered as 100%.

The effect of competing substrate HPP on isobutene production by the RnKICD wild-type and F336V mutant was determined using 10 or 1000µM of HPP. Isobutene production was observed to be reduced for both wild-type (~12% or 95% reduction) and F336V mutant (~24% or 95% reduction) in presence 10 or 1000µM of HPP, respectively. Although the F336V mutant displayed negligible consumption of HPP, the inhibition assay suggests that HPP still displays competition to the KIC thus hindering the isobutene production rates.

**
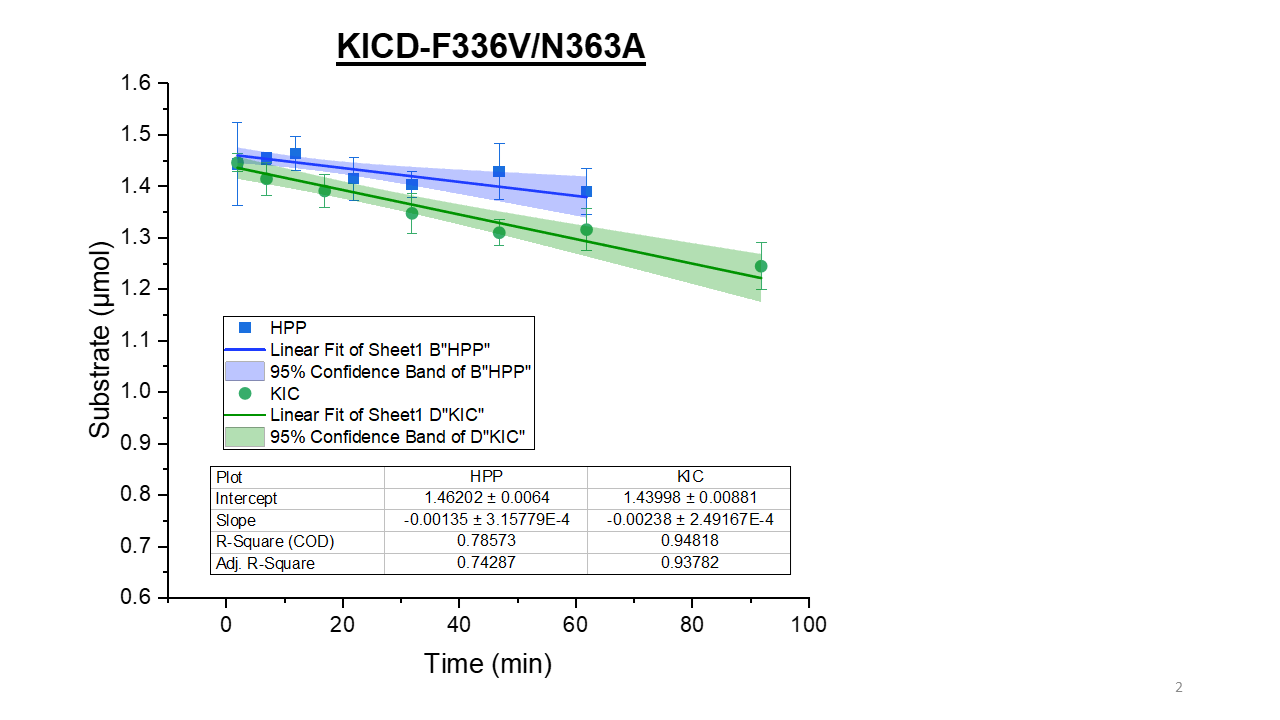

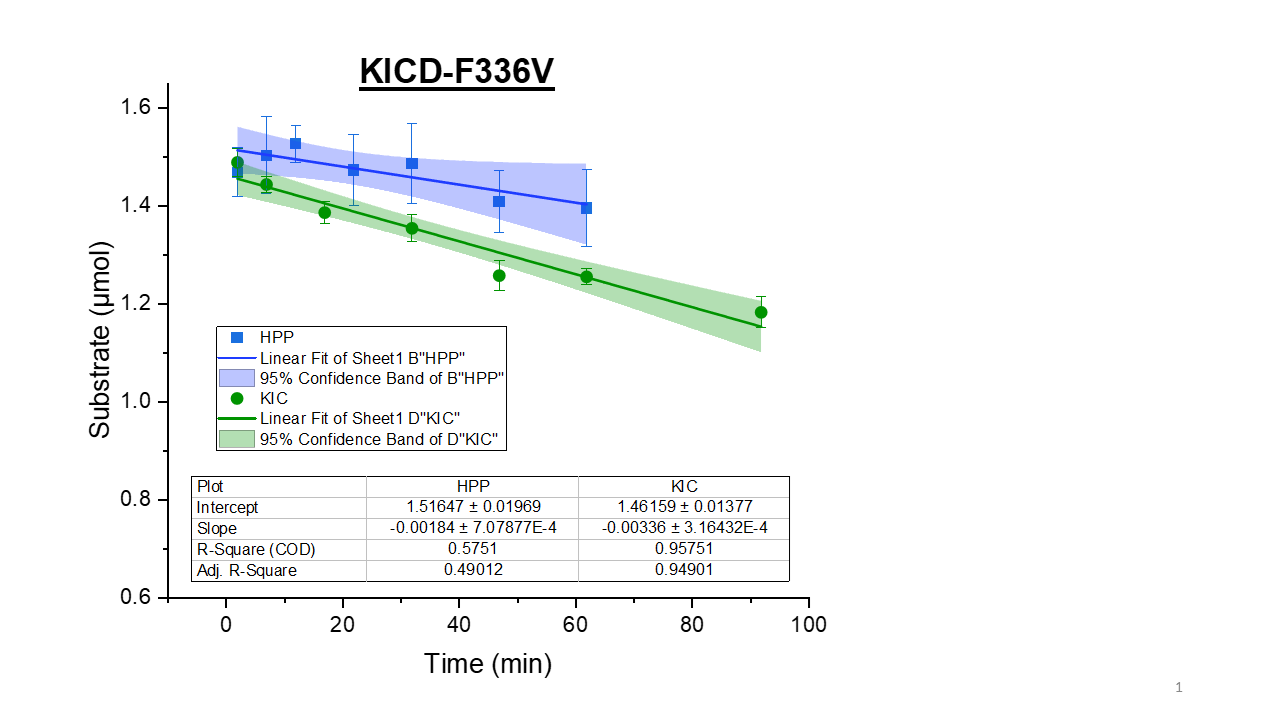

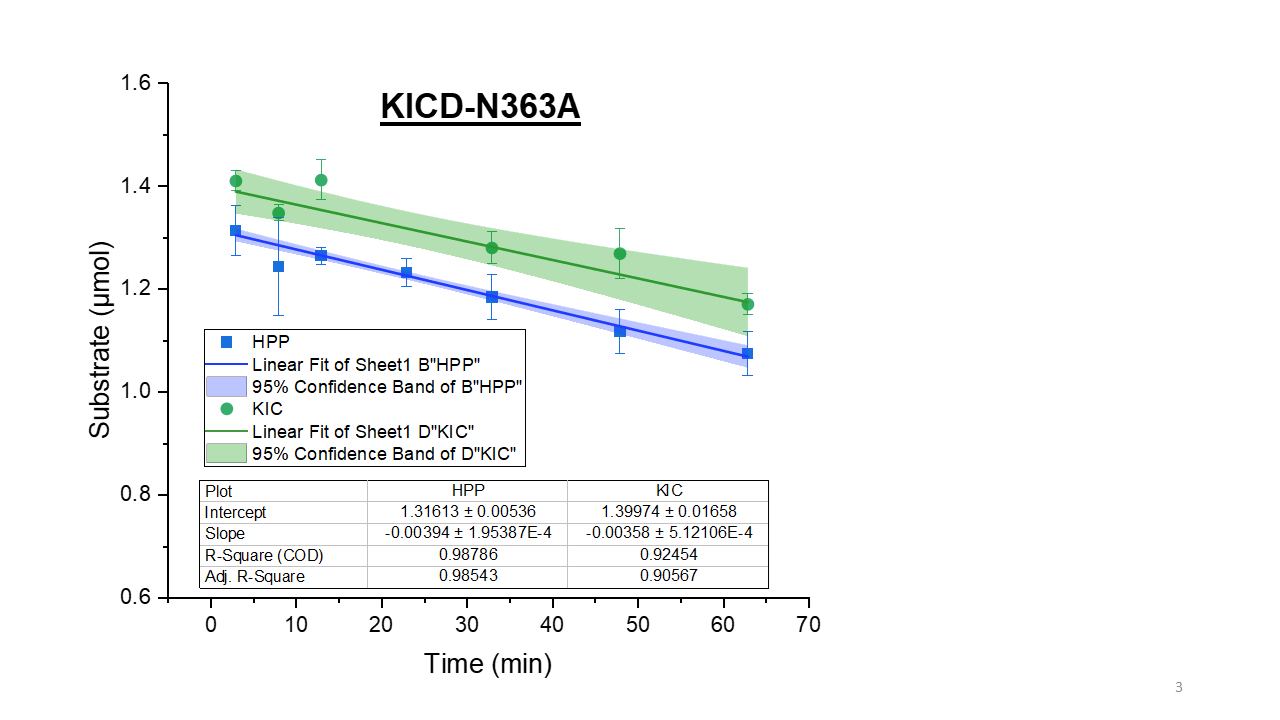

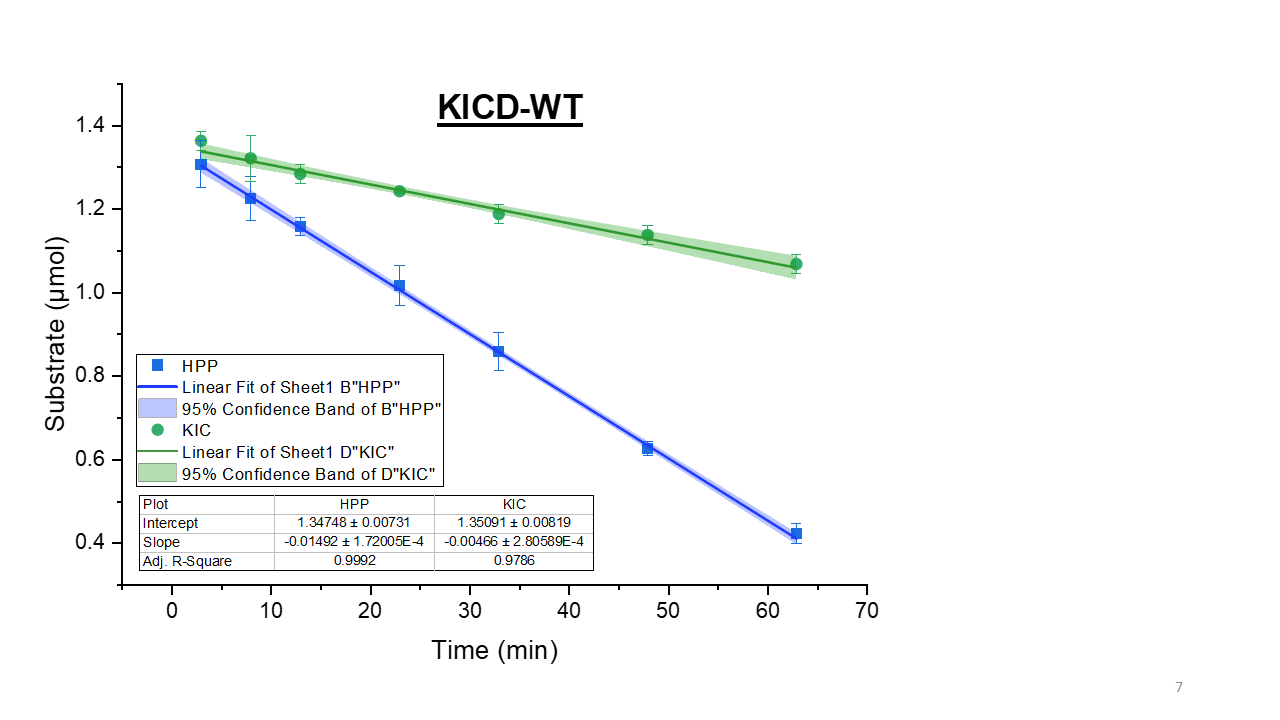
Supplementary Figure 5**

**F**

**E**

**D**

**C**

**A**

**B**


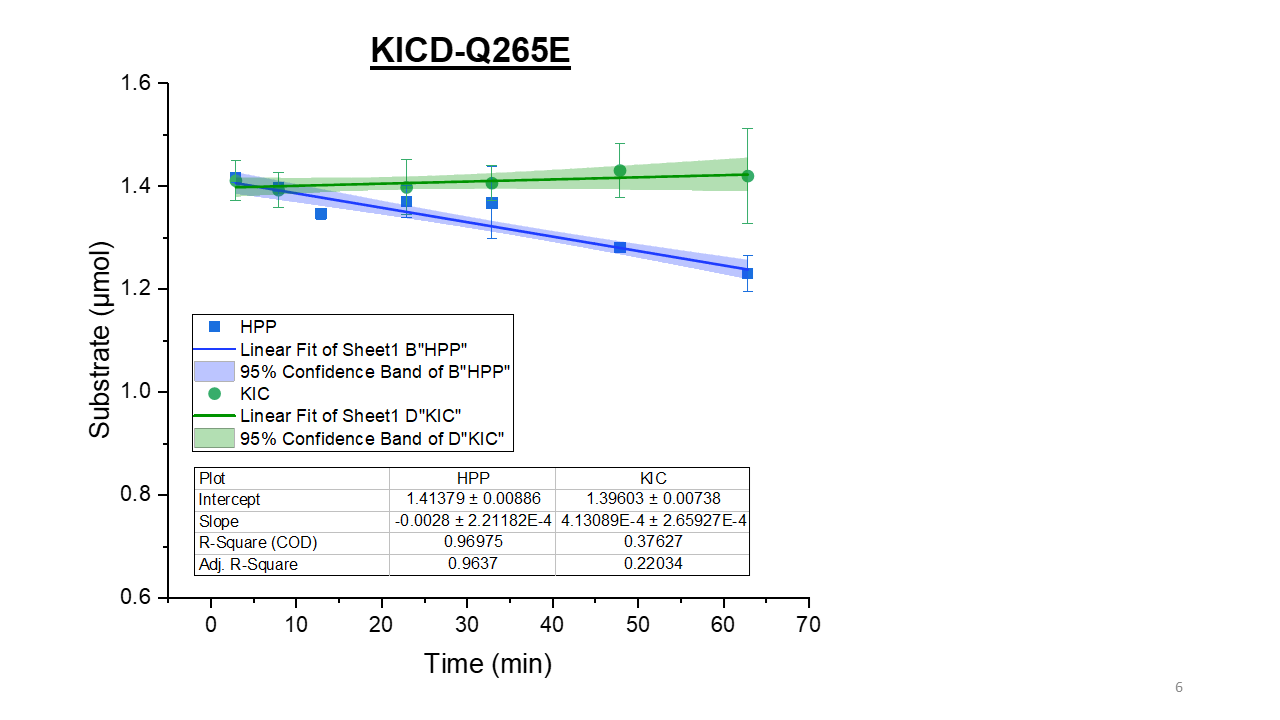

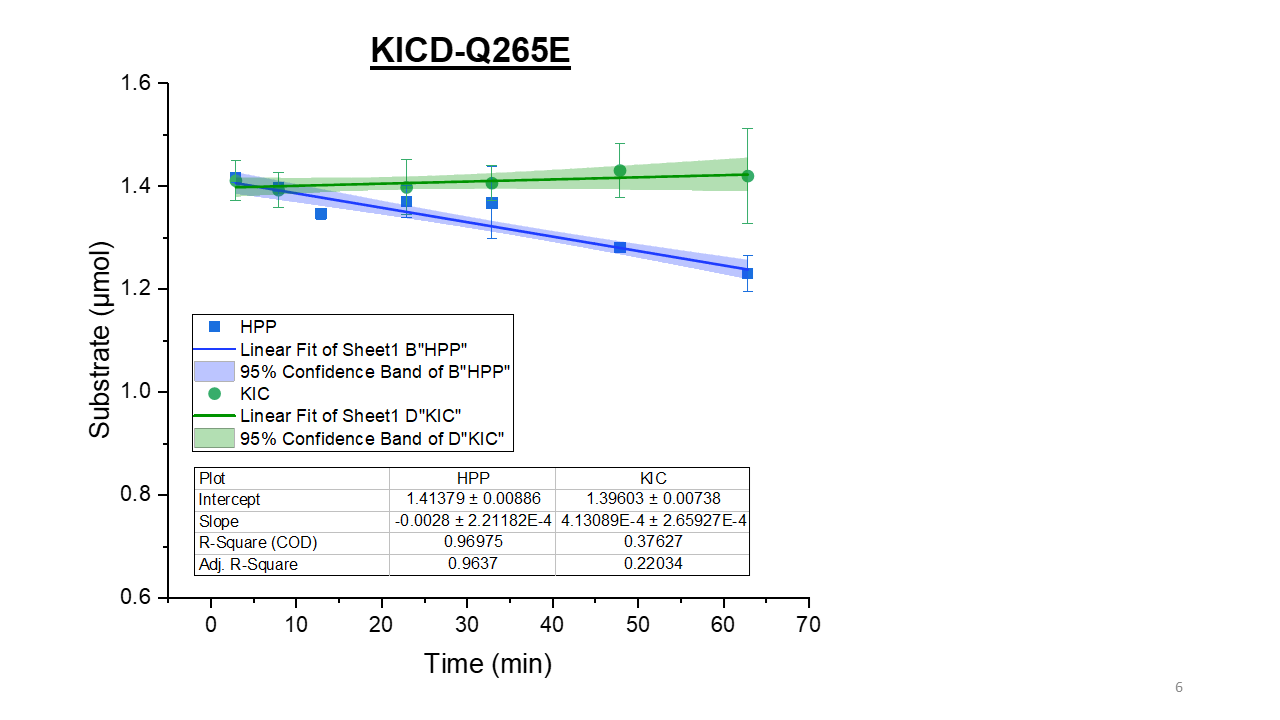

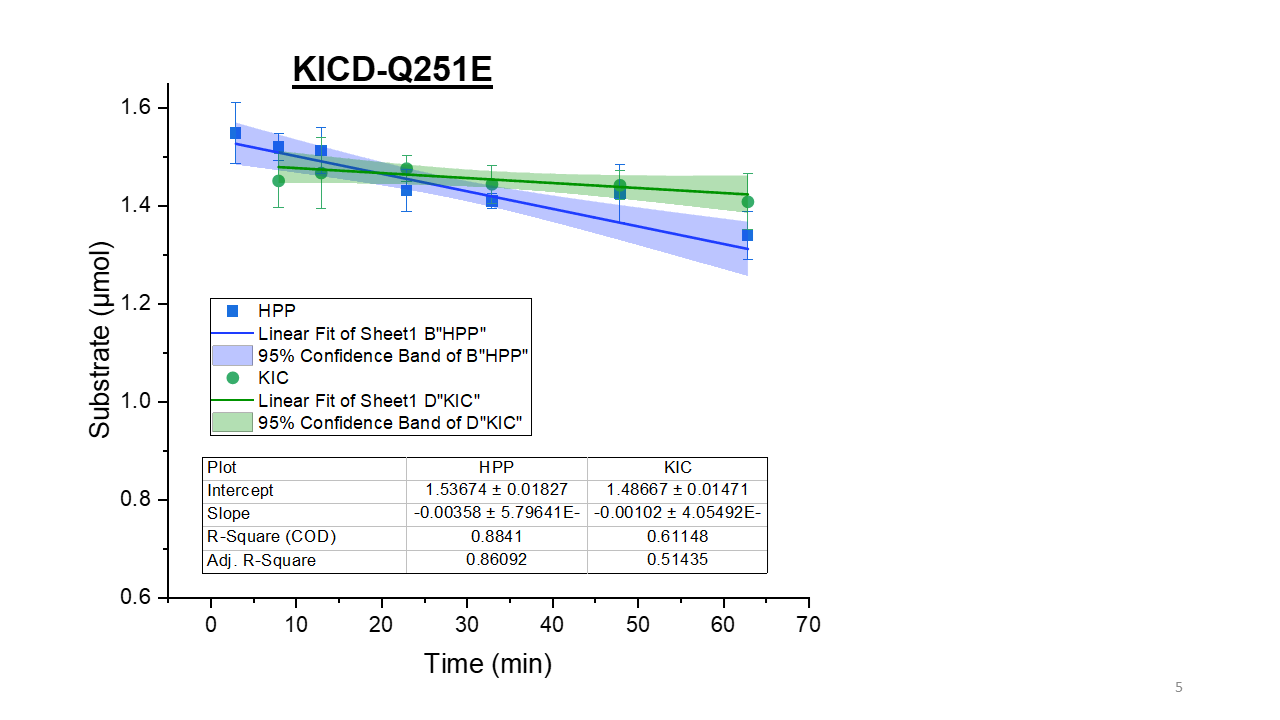

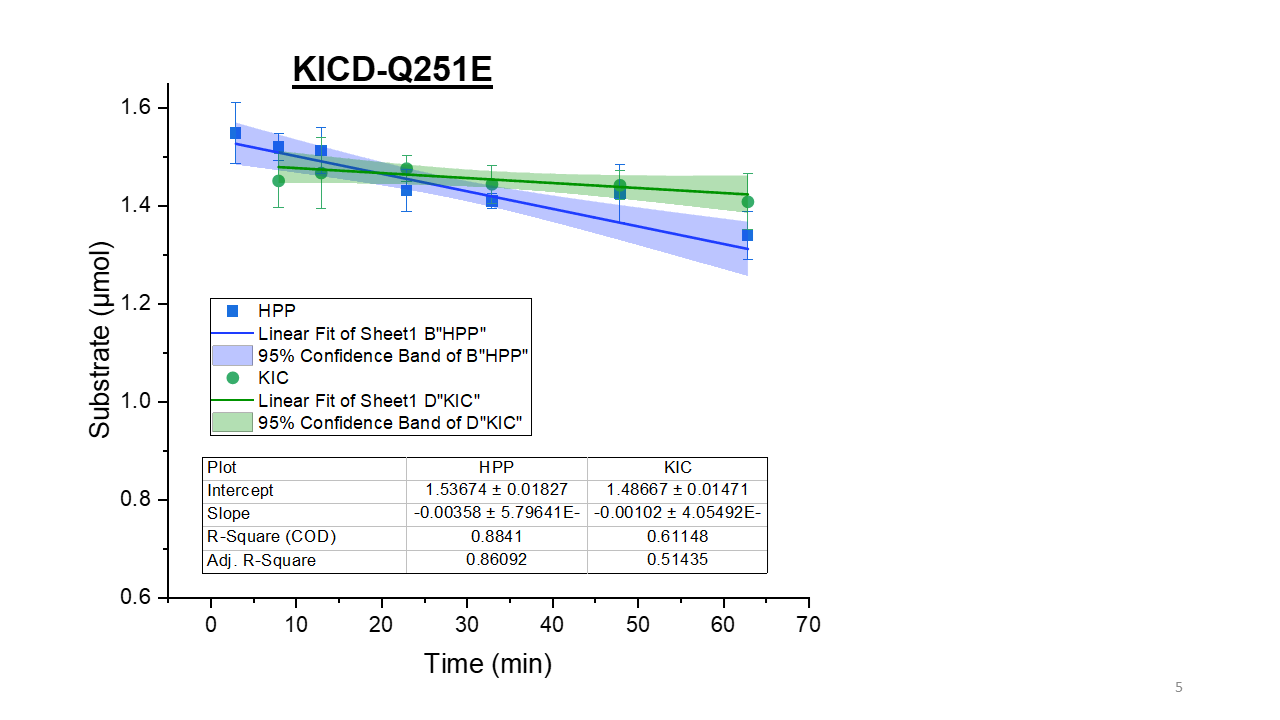


**G**

**
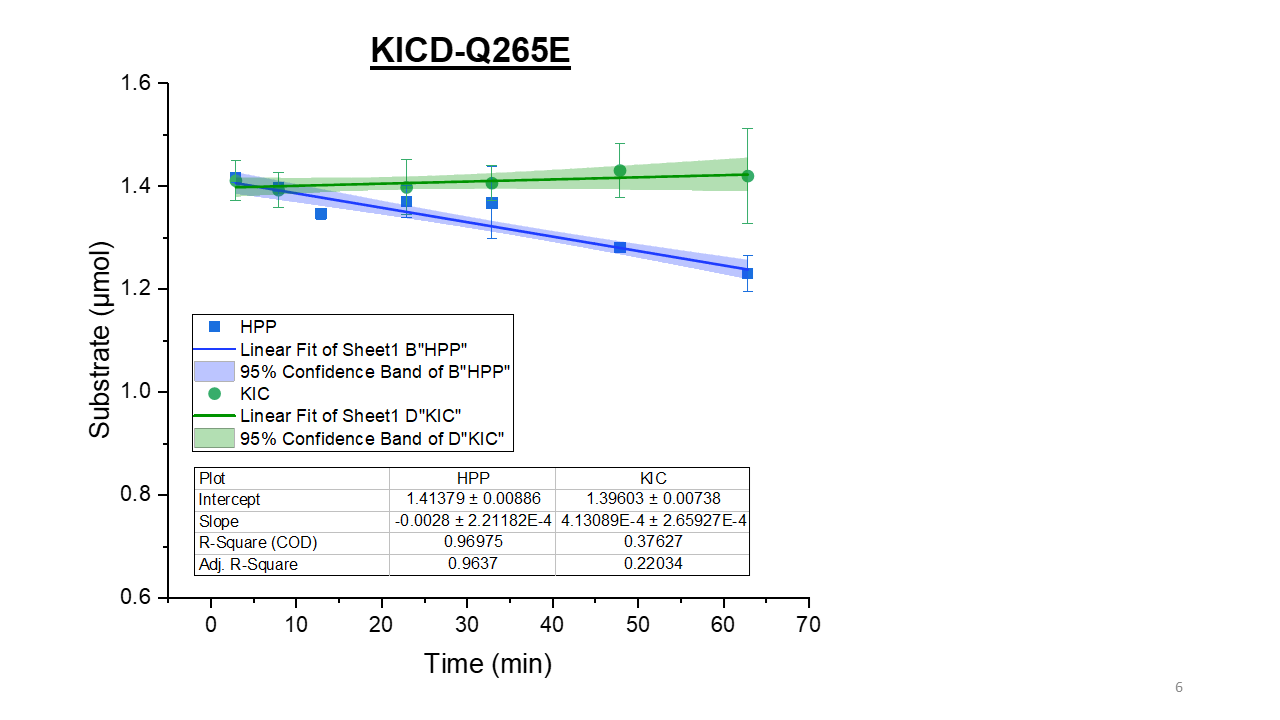
**

**Supplementary Figure 5**. Progress curves of HPP (blue squares) and KIC (green circles) consumption of purified *Rn*KICD variants at 30°C using DNPH assay. Each point represents mean and standard deviation of three technical replicates containing 4.2 µM enzyme , 2.5 mM substrate (either KIC or HPP), 0.5 mM FeSO_4_, 0.5 mM sodium ascorbate, and 1 mM dithiothreitol in the reaction buffer (10 mM MES, 150 mM NaCl, pH 6.0). The substrate depletion rates and error (in µmol min^-1^) for HPP (blue line and shading) and KIC (green line and shading) are illustrated by linear regressions with 95% confidence bands. A. Substrate depletion of *Rn*KICD-WT. B. Substrate depletion of *Rn*KICD-Q251E. C. Substrate depletion of *Rn*KICD-Q265E. D. Substrate depletion of RnKICD-N363A. E. Substrate depletion of *Rn*KICD-F336V. F. Substrate depletion of *Rn*KICD-F336V/N363A. G. Substrate depletion without enzyme (negative control).

**Supplementary Figure 6**


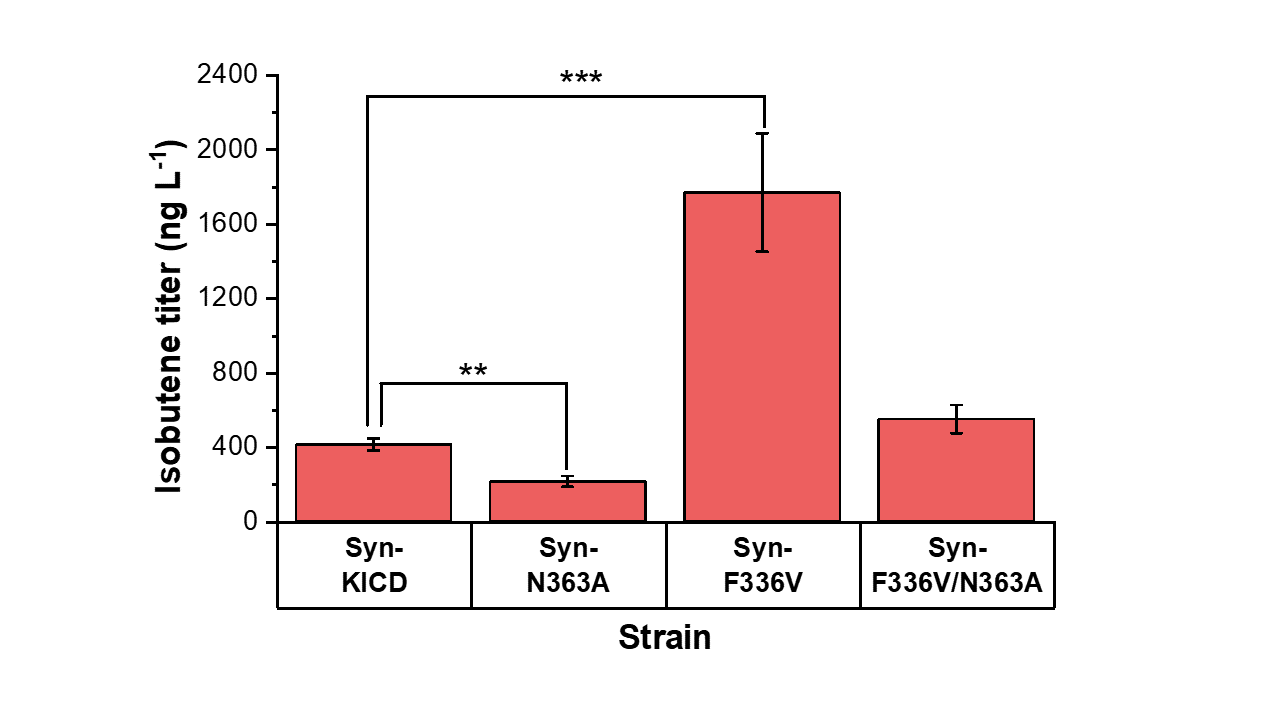


**Supplementary Figure 6**. Isobutene titer (ng L^-1^) by *Synechocystis* engineered strains after four days of batch cultivation. All the results represent the mean of five biological replicates; error bars represent the standard deviation. Asterisks represent significant differences between the corresponding strain and the base strain, ** p < 0.01, *** p < 0.001 in *t*-test.

**Supplementary Table 1** Primers used in this study for site-directed mutagenesis experiment.

| Name | Sequence 5'-3' | Used for |
| --- | --- | --- |
| **pETBB_BB1_FW** | CAAGCGCTCATGAGCCCGAA | General Sequencing |
| **pETBB_BB_RV** | TCGCCAATCCGGATATAGTTCC |  |
| **N363A_FW** | CGCCGGCgctTTTAATTCCTTATTCAAGGCCTTTGAG | N363A mutagenesis |
| **N363A_RV** | GAATTAAAagcGCCGGCGCCAAATCCTTGG |  |
| **Q251E_FW** | GAAAAAATCCgagATTCAAGAGTATGTTGATTATAACGG | Q251E mutagenesis |
| **Q251E_RV** | CTTGAATctcGGATTTTTTCCGGCCAG |  |
| **Q265E_FW** | CGTAgagCATATCGCCCTGCGTACCGAAG | Q265E mutagenesis |
| **Q265E_RV** | GATATGctcTACGCCGGCGCCAC |  |
| **F336V_FW** | GCAAATCgTTACTAAACCCATGCAGG | F336V mutagenesis |
| **F336V_RV** | GTTTAGTAAcGATTTGCAGCAGATAGCCC |  |

**Supplementary Table 2** Primers used in this study for iterative saturation mutagenesis experiment.

| Name | Sequence 5'-3' | Used for |
| --- | --- | --- |
| **ISM_F336_NDT_FW** | /5Phos/CNDTACTAAACCCATGCAGGACC | Generation of F336 site-saturation mutagenesis library |
| **ISM_F336_RV** | /5Phos/ATTTGCAGCAGATAGCCCTTTTC |  |
| **ISM_F336_TGG_FW** | /5Phos/CTGGACTAAACCCATGCAGGACC |  |
| **ISM_F336_VHG_FW** | /5Phos/CVHGACTAAACCCATGCAGGACC |  |
| **ISM_Q334_FW** | /5Phos/CTTTACTAAACCCATGCAGGACC | Generation of Q334 site-saturation mutagenesis library |
| **ISM_Q334_FW** | /5Phos/ATAHNCAGCAGATAGCCCTTTTCATC |  |
| **ISM_Q334_TGG_RV** | /5Phos/ATCCACAGCAGATAGCCCTTTTC |  |
| **ISM_Q334_VHG_RV** | /5Phos/ATCDBCAGCAGATAGCCCTTTTC |  |
| **ISM_seq_Q334_F336_FW** | ATTACCACTATTCGCCATCTGCGC | Library Sequencing |

**Supplementary Table 3** Comparison of in vitro KIC consumption rate at 2.5 mM substrate expressed as specific activity (U mg^-1^) and isobutene production rate (ng mg^-1^ min^-1^) at 3 mM KIC also expressed as specific activity (U mg^-1^). U = µmol min^-1^. Ratio was calculated by dividing the isobutene production rate by KIC consumption rate.

|  | **RnKICD (WT)** | **N363A** | **F336V** | **F336V/ N363A** |
| --- | --- | --- | --- | --- |
| KIC consumption rate (U mg^-1^) | 0.035 ± 0.0023 | 0.030 ± 0.0034 | 0.024 ± 0.0029 | 0.016 ± 0.0021 |
| Isobutene production rate (U mg^-1^) | 0.00127 ± 0.00012 | 0.00110 ±  0.00004 | 0.00080 ± 0.00004 | 0.00053 ± 0.00003 |
| Ratio (%) | **3.7 ± 0.4** | **3.7 ± 0.4** | **3.3 ± 0.3** | **3.3 ± 0.4** |

**Supplementary Material 1:** Coding sequence of N-terminal-StrepII tagged RnKICD with StrepII highlighted in blue font and glycine-serine linker ([G-S]_3_) highlighted in red font.

ATG**TGGAGTCATCCTCAGTTCGAGAAGGGTAGCGGAAGTGGATCT**ATGACTACCTATTCCAACAAGGGACCAAAACCAGAACGGGGGCGTTTTCTCCACTTTCATTCCGTGACTTTTTGGGTTGGTAATGCCAAGCAGGCCGCGAGCTTCTATTGCAACAAAATGGGCTTTGAACCCTTAGCTTATAAAGGCTTGGAAACTGGTAGCCGCGAAGTGGTGAGTCACGTTATCAAACAAGGAAAGATCGTCTTTGTTCTCTGTTCTGCCTTGAATCCCTGGAATAAAGAAATGGGTGATCATCTCGTAAAACACGGAGATGGAGTTAAAGACATTGCCTTCGAAGTGGAAGACTGCGAACATATTGTCCAAAAGGCGCGTGAACGCGGTGCGAAAATTGTTCGAGAGCCATGGGTGGAAGAGGACAAGTTTGGAAAAGTAAAATTTGCCGTGCTTCAAACTTACGGCGATACCACCCATACCCTCGTAGAAAAAATTAATTACACTGGGCGATTTCTGCCGGGCTTTGAAGCCCCCACCTATAAGGATACTTTATTACCCAAGTTGCCATCTTGTAATTTAGAAATTATTGACCATATTGTGGGTAATCAGCCAGATCAGGAAATGGAATCCGCGAGTGAGTGGTACTTAAAAAATTTACAGTTCCATCGGTTTTGGAGTGTGGATGATACCCAGGTGCATACCGAGTACAGTAGTCTCAGGAGCATTGTTGTGGCGAATTATGAAGAATCCATCAAAATGCCGATTAATGAGCCTGCTCCTGGCCGGAAAAAATCCCAAATTCAAGAGTATGTTGATTATAACGGTGGCGCCGGCGTACAGCATATCGCCCTGCGTACCGAAGATATTATTACCACTATTCGCCATCTGCGCGAACGCGGCATGGAATTTTTAGCCGTTCCAAGCAGTTATTATCGTCTCCTACGTGAAAACTTAAAAACTTCCAAGATCCAAGTGAAGGAGAATATGGATGTTCTCGAAGAATTAAAAATCCTTGTGGATTATGATGAAAAGGGCTATCTGCTGCAAATCTTTACTAAACCCATGCAGGACCGGCCCACTTTGTTTTTAGAGGTGATCCAGCGTCATAATCACCAAGGATTTGGCGCCGGCAATTTTAATTCCTTATTCAAGGCCTTTGAGGAAGAACAAGCCTTACGTGGCAATTTGACTGACTTAGAGACCAACGGCGTGAGATCCGGTATGTAA
